# The effect of physiographic and hydrologic complexities and their alterations on the distribution of obligate freshwater dolphins

**DOI:** 10.1002/ece3.10106

**Published:** 2023-05-21

**Authors:** Anu Rai, Tawqir Bashir, Elio Guarionex Lagunes–Díaz, Bibek Shrestha

**Affiliations:** ^1^ Department of Environmental Science and Engineering, School of Science Kathmandu University Dhulikhel Nepal; ^2^ Sustainability and Environmental Studies Endeavor (SENSE) Kathmandu Nepal; ^3^ River Dolphin Trust Kailali Nepal; ^4^ Centre of Research for Development University of Kashmir Srinagar India; ^5^ Instituto de Ecología Veracruz Mexico; ^6^ Yale School of the Environment New Haven Connecticut USA

**Keywords:** Amazon dolphin, barrages, biodiversity, dams, Ganges dolphin, hydrologic alterations, hydrologic complexities, Indus dolphin, obligate freshwater dolphins, review

## Abstract

Physiographic and hydrologic complexities play major role in determining the habitat suitability for river dolphins. However, dams and other water development structures alter hydrologic regimes that degrade habitat conditions. For the three extant species of obligate freshwater dolphins, namely: Amazon dolphin (*Inia geoffrensis*), Ganges dolphin (*Platanista gangetica*), and Indus dolphin (*Platanista minor*), the threat is high as dams and water‐based infrastructure dotted throughout their distribution range impact dolphin populations by restricting their movement. But there is also evidence of localized increase in dolphin population in certain segments of habitats affected by such hydrologic alterations. Hence, the impacts of hydrologic alterations on dolphin distribution are not as binary as it seems. We aimed to ascertain the role of hydrologic and physiographic complexities in determining the distribution of the dolphins in their geographic ranges using density plot analysis and also to understand how hydrologic alterations in the rivers affect their distribution using a combination of density plot analysis and review of literature. The influence of some of the study variables such as distance to confluence and sinuosity was similar across species—for instance, all three dolphin species preferred slightly sinuous river segments and habitats near confluences. However, varying influences across species were observed for some other variables such as river order and river discharge. We assessed 147 cases of impacts of hydrological alterations on dolphin distribution by categorizing the reported impacts in nine broad types out of which habitat fragmentation accounted for the majority of the impacts (35%) followed by habitat reduction (24%). These endangered species of freshwater megafauna will experience further intensified pressures as more large‐scale hydrologic modifications such as damming and diversion of rivers are underway. In this context, basin‐scale water‐based infrastructural development planning should take into consideration the salient ecological requirements of these species to ensure their long‐term survival.

## Graphical abstract

All the three surviving obligate freshwater dolphins are Endangered due to continuous decline in population and distribution range. Our study helps to ascertain the role of hydrologic and physiographic complexities on determining the distribution of dolphins and also discusses how the alterations induce change in their distribution. This will prove useful as hydrologic alterations are planned throughout the range of the species.

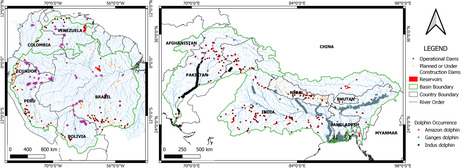

## 1 INTRODUCTION

Physiographic and hydrologic complexities play a major role in maintaining the ecological dynamics of rivers (Poff et al., 1997), making riverine habitats suitable for apex predators such as dolphins (Reeves & Leatherwood, 1994). River dolphins show a preference for specific hydraulic nodes such as confluences, meanders, and deep pools, which provide refuge from hydraulic forcing combined with higher biological activity (Smith, 1993; Smith et al., 2009; Smith & Mansur, 2012). Alterations in the hydrologic regime of rivers caused by dams, barrages (low‐gated dams for water diversion rather than storage), and water abstraction for irrigation and power generation are known to impair riverine ecosystems globally by degrading habitat conditions (Aggarwal et al., 2020; Poff et al., 2010). As hydrophysical parameters are major determinants of dolphin distribution and abundance, their modifications through interactive effects of flow regulation can potentially deteriorate dolphin habitat by decreasing the overall carrying capacity of the rivers (Huang et al., 2012; Paudel et al., 2020). The three extant species of obligate freshwater dolphin—Ganges dolphin (*Platanista gangetica*), Indus dolphin (*Platanista minor*), and Amazon dolphin (*Inia geoffrensis*)—are impacted by the threats relating to the modification of hydrologic regimes and degradation of riverine ecosystems. The fourth obligate freshwater dolphin—Yangtze dolphin (*Lipotes vexillifer*)—has been deemed possibly extinct (Smith et al., 2020). The stressors are becoming more prominent with an increasing number of flow‐modifying structures and water‐based infrastructure such as dams and barrages being developed and/or under planning throughout the distribution ranges of the dolphin species.

As river cetaceans including dolphins have similar habitat requirements and shared sensory and morphological characteristics (Campbell et al., 2022), the influence of some hydrophysiographic variables on dolphin occurrences may generally be similar across freshwater dolphin species. For example, confluences are preferred by Amazon dolphins and Ganges dolphins as they are associated with stable flows and fish aggregation (Martin & da Silva, 2004; Smith et al., 2009). Meanders tend to have higher nutrient content and also a prevalence of eddy countercurrents, which can disorient fishes, thus facilitating predation by dolphins (Guizada & Aliaga‐Rossel, 2016; Mazumder et al., 2014). Deep pools are known to facilitate longer dives and hence yield the highest usable area for dolphins (Paudel et al., 2021). A combination of deep and shallow river segments also supports activities such as resting, feeding, and reproduction (Mosquera‐Guerra et al., 2020; Sinha & Kannan, 2014).

Hydrologic alterations by flow‐modifying structures including dams, barrages, canals, irrigation diversions, embankments, loop cutting, spur dikes, and/or activities such as dredging have been reported to generally impact freshwater dolphins as they change the hydrological complexities of the riverine ecosystems. For instance, hydrologic alterations have restricted the movement of dolphins by affecting longitudinal and lateral habitat connectivity, rendering them isolated into several subpopulations (Braulik & Smith, 2019; Smith et al., 2000). Straightening of river channels affects meanders (Sinha, 2000), and water abstraction can impact tributary flow and cause a reduction in floodplain connectivity (Kelkar et al., 2010). Likewise, dredging can affect dolphin habitat physically and acoustically (Dey et al., 2019). Dams and waterways both deeply impact flood pulse (Gomez‐Salazar et al., 2012).

While the decline in the freshwater dolphin population has been attributed to hydrologic alteration by water‐based infrastructures such as dams, there is also clear evidence of increase in population of dolphins in certain segments of rivers affected by such hydraulic structures. For instance, the Bijnor—Narora stretch of the Upper Ganges, the Upper Ganges Ramsar Site, and the Katarniaghat Wildlife Sanctuary in Uttar Pradesh, India, have witnessed increases in Ganges dolphin population mostly due to increase in water depth in the rivers (Kreb et al., 2010; Sinha & Kannan, 2014). An increase in sinuosity in certain sections of the Ganges river after the construction of Bijnor barrage has led to additional hydraulic refuge for dolphins (Sonkar & Gaurav, 2020). Moreover, canals can also provide refuges for feeding and mating on a short‐term basis and can increase the aggregate habitat while providing greater refuge during anthropological disturbance (Prajapati, 2021; Smith et al., 2001). Besides, other structures such as groins and spur dikes result in the creation of artificial eddy countercurrent habitats (Smith, Aminul Haque, et al., 1998), which dolphins utilize for predation of fish. Hence, the impacts of flow alterations in riverine habitats are not as binary as they seem to be for river dolphins.

With this background, we hypothesize that the dolphin distributions will show an overall similar pattern in relation to hydrologic and physiographic parameters across species of obligate freshwater dolphins, with an overall variable influence of hydrological alterations on distributions varying both in direction and in contribution with respect to species. We therefore aim to first ascertain the role of hydrologic and physiographic complexities of rivers in determining the distribution of the extant obligate freshwater dolphin species and then discuss the changes in dolphin distributions induced by alterations in flow regimes. To this end, we have carried out a review of the research conducted on this phenomenon, and we have analyzed dolphin distribution for the entire ranges of the three dolphin species with an emphasis on the probable impacts of existing and proposed dams.

## 2 METHODS

To ascertain the effect of physiographic and hydrologic complexities as well as variables relating to hydrologic alteration, we performed density plot analysis of presence‐only data of the species plotted along variables. We also carried out a review of literature to assess the impacts of dams and other water‐based infrastructures on obligate freshwater dolphins.

### 2.1 Occurrence records

**FIGURE 1:**
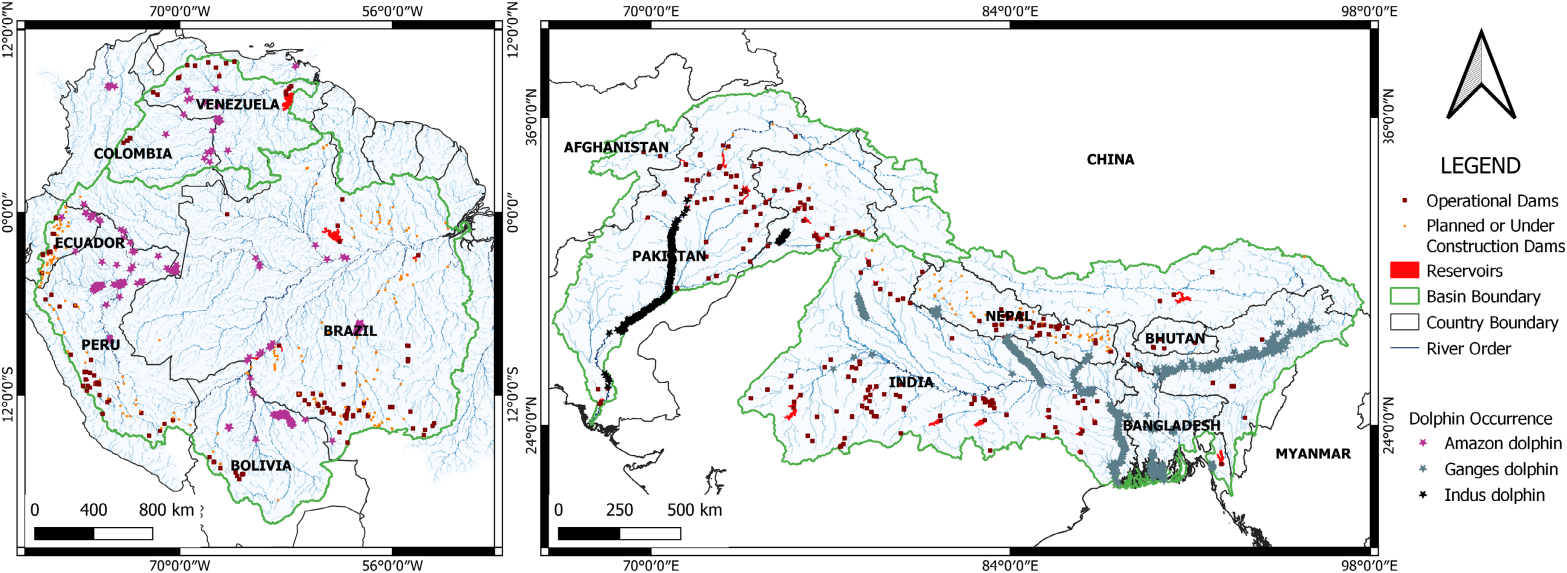
Occurrence records of three obligate freshwater dolphin species in their entire distribution ranges compiled from records reported by different researchers, personal collection, and online repositories.

We compiled the occurrence records for the dolphin species across their entire ranges of distribution to obtain presence‐only records of the species—Ganges–Brahmaputra–Meghna river basin and the Karnaphuli–Sangu river basin henceforth known as GBMKS basins along with the Ganges Delta in Bay of Bengal spanning Nepal, India, and Bangladesh for the Ganges dolphin, Indus river system in India and Pakistan for the Indus dolphin, and Orinoco and Amazon river systems in Bolivia, Brazil, Colombia, Ecuador, Peru, and Venezuela for the Amazon dolphin (Figure 1). We discarded the occurrence records explicitly mentioning Araguaian River Dolphin (*Inia araguaiaensis*) as its taxonomic status is not completely resolved and needs further evaluation (da Silva et al., 2018).

Our compilation of the occurrence records of the dolphins included reports by researchers in several studies (see Supporting Information Annex I), personal collection of one of the authors (TB), and online repositories—GBIF (https://www.gbif.org/) (GBIF.org, 2022a, 2022b) and OBIS‐SEAMAP (http://seamap.env.duke.edu/). The observations withheld or with imprecision greater than 1000 m and stated records prior to 1995 were discarded from the GBIF data. The duplicates of the records were also discarded. This yielded 965 presence records of the Ganges dolphin, 403 presence records for the Indus dolphin, and 925 presence records for the Amazon dolphin. We then used these presence records for density plot analysis of the environmental variables that determine the distribution of the dolphins. Majority of these records were recent with more than 35% recorded after 2017.

### 2.2 Environmental variables

The data on environmental variables were obtained from HydroSHEDS (https://hydrosheds.org/) and RiverATLAS/HydroSHEDS (https://www.hydrosheds.org/page/hydroatlas) (Linke et al., 2019). HydroSHEDS provides a series of gridded datasets from where we obtained hydrologically conditioned Digital Elevation Model (DEM) and flow accumulation data. RiverATLAS/HydroSHEDS is a global compendium of hydro‐environmental variables in vector format, which have been derived from existing datasets. The data on these variables are freely available as river network lines. It comprises 56 hydro‐environmental variables such as natural discharge consisting of 281 individual attributes such as the variable natural discharge represented in three forms—annual average, annual mean, and annual maximum along with 14 attributes of HydroRivers such as river order (Linke et al., 2019).

We have used 14 variables as physiographic and hydrologic variables while two other variables—degree of regulation and irrigated area extent—represent alterations (Table 1). For use in density plot analysis, the variables must be in raster form. Hence, the variables which were not available in raster format were rasterized with the v.to.rast module in GRASS GIS 7.8.4 at 30 arc seconds spatial resolution. We resampled hydrologically conditioned DEM from 3 arc second resolution to 30 arc seconds to make it compatible with other environmental layers. As the study species are exclusively aquatic, we clipped all the environmental variable layers by river networks for use as input layers.

**TABLE 1:** Physiographic and hydrologic variables and variables representing alterations used in the study.

Variables	Description	Process of obtaining variables	Observed range
Annual average discharge	Annual average discharge estimates based on long‐term (1971–2000) average “naturalized” discharge and runoff values	Extracted from RiverATLAS/HydroSHEDS	0–230,000 m^3^/sec
Flow accumulation	The extent of upstream area (in number of cells) draining into each cell	Extracted from HydroSHEDS	1–696,575
Hydrologically conditioned digital elevation model	DEM whose flow direction defines the expected flow of water over the terrain	Extracted from HydroSHEDS	(−185) – 8527 m (at 3 arc second)
Stream gradient	The ratio of the elevation drop within the river reach (i.e., the difference between min. and max. elevation along the reach) and the length of the reach.	Extracted from RiverATLAS/HydroSHEDS	0–20,0000 decimeter/km
Classical ordering of river	River order following classical ordering system such that order 1 corresponds to main channel, order 2 corresponds to tributaries that flow into order 1 and so on.	Extracted from RiverATLAS/HydroSHEDS	1–13
River flow corresponding to logarithmic size class	River order using flow to distinguish logarithmic size classes such that order 1 corresponds to river reaches with long‐term average discharge ≥100,000 m^3^/s; order 2 represents discharge ≥10,000 m^3^/s and so on.	Extracted from RiverATLAS/HydroSHEDS	1–10
Strahler order	River order following Strahler ordering system that defines stream order on the basis of hierarchy of tributaries such that when streams of same order join the resulting stream has one higher order.	Extracted from RiverATLAS/HydroSHEDS	1–10
Distance to confluence	Distance of a raster cell to the nearest confluence	Calculated from river networks lines	0–60541.9 m (Only for the extent studied)
River area	The river area along river reach (channel width × length of the reach)	Extracted from RiverATLAS/HydroSHEDS	0–4,300,000 ha
River volume	The river volume along river reach (channel width × depth × length)	Extracted from RiverATLAS/HydroSHEDS	0–300,000 million m^3^
Depth	Depth along river reach (river volume/ river area)	Calculated from river volume and river area variables from RiverATLAS/HydroSHEDS	0–25.44 m (Only for the extent studied)
Sinuosity	The ratio of actual stream length to shortest path length possible for the stream	Calculated from river network lines	1–7.64 (Only for the extent studied)
Inundation extent—annual minimum	Permanently inundated extent	Extracted from RiverATLAS/HydroSHEDS	0%–100%
Inundation extent—annual maximum	Seasonally inundated extent	Extracted from RiverATLAS/HydroSHEDS	0%–100%
Degree of regulation	Index of how strongly a dam or a set of dams can affect the natural flow regime of downstream river reaches	Extracted from RiverATLAS/HydroSHEDS	0%–1000%
Irrigated area extent	Irrigated area extent in total watershed upstream of reach pour point	Extracted from RiverATLAS/HydroSHEDS	0%–100%

The physiographic and hydrologic variables used here are important factors determining the distribution of dolphins. Confluences are preferred as they enable stable flows and fish aggregation, meanders enable prevalence of eddy countercurrents, and feature higher nutrient content while deep pools enable higher usable areas. Whereas alterations in the form of degree of regulation and irrigated area extent restrict the movement of dolphins and render population isolation.

Depth along river reach was calculated by dividing river volume by river area both of which were obtained from RiverATLAS/HydroSHEDS. For calculating sinuosity, we first built polylines from the river segments with v.build.polyline and then we split those segments with v.split module both using GRASS GIS with a maximum segment length of 5000 m. This segment length was also chosen by Smith et al. (2008) for determining sinuosity as habitat selection variables for freshwater cetaceans. Finally, we calculated sinuosity using the v.to.db module. For the calculation of distance to confluence, we used line intersection to identify the location of confluences. We then employed gridDistance function in software program R (RStudio 2022.02.3) to calculate the distance to cells with the omission of land feature (Hijmans et al., 2023).

### 2.3 Density plot analysis

To ascertain the physiographic and hydrologic complexities pertaining to dolphin distribution represented by presence locations of the species, we created density plots of species presence‐only data and physiographic and hydrologic variables: discharge, flow accumulation, stream elevation, stream gradient, stream order, distance from confluence, river volume, river area, depth, sinuosity and inundation extent as well as variables that show the effect of hydrologic alterations: degree of regulation and irrigated area extent. We also performed two‐sample z‐test to test the statistical significance of the difference between the values of variables associated with dolphin occurrence locations and the overall values for the overall study area at 95% confidence interval.

The habitat preference of dolphins noted in earlier studies is based on observations, which are mainly qualitative in nature. For instance, the preference for confluences by dolphins has been noted by many studies (Bashir et al., 2010; Sinha & Kannan, 2014). This study provides a quantitative account of such habitat preferences.

### 2.4 Data compilation on hydrological alterations

For assessing the impacts of dams and other water‐based infrastructures on obligate freshwater dolphins, we searched Google Scholar for studies stating the site‐specific impacts on dolphin distribution that have been observed, speculated, or predicted due to hydrological alterations. Generic accounts of impacts such as restriction of dolphin movement by dams or fragmentation of habitat of freshwater cetacean by barrages were filtered out. A combination of specific keywords or their derivatives: hydrologic complexities, habitat fragmentation, canal, dam, barrage, water diversion, and flow variation + Ganges dolphin, Indus dolphin, South Asian dolphins, Amazon dolphin, South American dolphin or freshwater dolphins were used to search published articles for review. This search was supplemented with articles citing or cited by those literatures along with relevant studies from our own collection. In the case a research article cited another study, the original articles were sought wherever possible. Overall, we collected data from 45 articles for our analysis in this review (Supporting Information Annex II).

The impacts of hydrological alterations noted in the literature were classified into nine broad categories: competition, creation of suitable habitat condition, decline in food availability, extirpation, fishery overlap, habitat fragmentation, habitat reduction, pollution, and range reduction (Table 2).

**TABLE 2:** Categorization of impacts caused by alterations to water courses.

Impact	Broad categorization of impact	Positive, negative, mixed impact	Observed, speculated, or predicted impact	Direct or indirect impact
Erroneous construction of the fish bypass allowing upstream migration of *Inia geoffrensis* affecting Bolivian dolphin (*Inia boliviensis)* population (Tavera et al., 2010)	Competition	Negative	Predicted	Indirect
Impoundments upstream of Narora barrage maintaining suitable habitat condition for Ganges dolphin (Sonkar & Gaurav, 2020)	Creation of suitable habitat condition	Positive	Observed	Direct
Decline in fish likely due to interference with spawning cycle of migratory fishes due to construction of dam in Kailashpuri (Smith, 1993)	Decline in food availability	Negative	Speculated	Direct
Extirpation of dolphins from Mahakali river in Nepal (Smith et al., 2000)	Extirpation	Negative	Observed	Direct
Irrigation diversion aggravating fishery impact in already impacted sites (Khanal et al., 2016)	Fishery overlap	Negative	Observed	Indirect
Indus dolphins split into five subpopulations (Braulik, 2006).	Habitat fragmentation	Negative	Observed	Direct
Shallowness due to water diversion obstructing dolphin movement (Kreb et al., 2010)	Habitat reduction	Negative	Observed	Direct
Water abstraction causing the decline of dilution capacity of rivers (Sinha et al., 2000)	Pollution	Negative	Observed	Indirect
Kaptai dam likely, significantly reducing range of Ganges dolphin (Aziz, 2019)	Range reduction	Negative	Speculated	Direct
The reservoir created by dam will probably be used by Bolivian river dolphin harboring large amounts of fish species but drastically reducing diversity affecting dolphins because of their wide feeding spectrum (Tavera et al., 2010)	Decline in food availability	Mixed	Predicted	Direct

Each impact was classified as positive, negative, or mixed. Positive impacts for instance include groins and spur dikes constructed for closing an old distributary channel resulting in creation of artificial eddy countercurrents (Smith, Aminul Haque, et al., 1998). Negative impacts include impacts such as reduction in upstream occurrence limit (Choudhary et al., 2012), whereas a mixed impact includes impact states such as reservoir creation harboring large amounts of fish species but drastically reducing fish diversity affecting dolphins because of their wide feeding spectrum (Tavera et al., 2010).

The type of hydrological alterations such as dams, barrages, loop cutting, and the rivers and countries where these alterations occurred were also noted. The Ganges river was further classified as Upper Ganges (from the origin till Varanasi), Middle Ganges (the segment between Varanasi and Rajmahal), and Lower Ganges (the section downstream of Rajmahal). The impacts were also broadly classified as being direct or indirect. For example, irrigation diversions aggravating fishing impacts on dolphins were considered an indirect impact (Khanal et al., 2016), whereas reduced flow caused by a hydrologic alteration represents direct impact on dolphins by reducing space availability, water velocity, and depth (Braulik et al., 2014).

The impacts were also classified as observed, speculated, or predicted impacts. Observed impacts include impacts based on observation statements such as, lack of observation of the dolphin in the Mahakali river flowing through Nepal due to lack of enough water in the upstream of the barrage (Smith et al., 1994). Impacts worded as “likely,” “could be,” “could have been”, for example, reported declines in fish diversity associated with flow regulation resulting in impact on river dolphin (Braulik et al., 2014) were classified as speculated impacts. Predicted impacts are the impacts for which construction of the hydrologic alteration has not been completed but for which impacts have been predicted, for example predicted exacerbation of mercury pollution due to flooding of surface area by Jirau Dam (Tavera et al., 2010).

## 3 RESULTS

### 3.1 Species distribution along hydrophysiographic variable gradients

**FIGURE 2:**
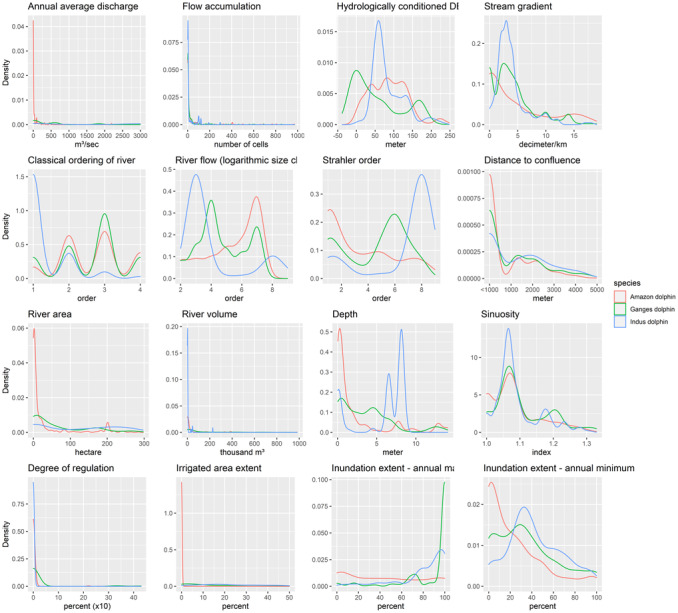
Density plots showing the distribution of presence‐only observation data of the Amazon dolphins, the Ganges dolphins, and the Indus dolphins along the gradients of hydrological and physiographic variables and the variables representing hydrologic alteration.

Dolphin distributions have shown variation along the gradients of physiographic and hydrologic variables (Figure 2, Tables 3 and 4). The dolphins were found to be distributed in a wide range of river discharge with the Indus dolphins mostly distributed in higher discharge areas than the Amazon dolphins or the Ganges dolphins. The same pattern also holds true for flow accumulation. The dolphin species also showed a preference for lower elevations in their distribution ranges. The presence of the Ganges dolphins in areas with negative hydrologically conditioned DEM denotes their presence in depression areas as well. These are areas that are surrounded by areas with higher elevation. The Amazon dolphins and the Ganges dolphins were observed in a wider range of stream gradients than the Indus dolphins.

**TABLE 3:** Range of values of hydrological and physiographic variables observed for 80 percentage reported dolphin occurrence.

Variables	Amazon dolphin	Ganges dolphin	Indus dolphin
Annual average discharge (m^3^/sec)	0–1000	0–500	250–5750
Flow Accumulation (number of cells)	0–25,000	0–25,000	25,000–1,025,000
Hydrologically conditioned Digital Elevation Model (m)	0–50	−25 – 175	45–135
Stream gradient (decimeter/km)	2.5–22.5	0–25	1–3
Classical ordering of river (order)	2–4	2–3	1–1
River flow corresponding to logarithmic size class (order)	5–7	3–7	3–3
Strahler order (order)	1–5	1–7	8–8
Distance to confluence (m)	250–1250	250–2250	250–2250
River area (ha)	0–25	25–175	25–725
River volume (million m^3^)	0–2500	0–5000	2500–42,500
Depth (m)	0.25–12.75	0.25–6.75	6.75–8.25
Sinuosity	1.025–1.125	1.05–1.25	1.01–1.17
Inundation extent—annual maximum (%)	2.5–97.5	72.5–97.5	77.5–97.5
Inundation extent—annual minimum (%)	2.5–47.5	2.5–97.5	2.5–72.5
Degree of regulation (%)	0–0.5	0–25	170–250
Irrigated area extent (%)	0–0.5	2.5–82.5	2.5–42.5

**TABLE 4:** Statistical significance of the difference between the values of variables associated with dolphin occurrence locations and the values for the overall study area (*p* < .05 denoted by *, *p*‐value < .01 denoted by **, and *p*‐value < .001 denoted by ***).

Variables	Amazon dolphin	Ganges dolphin	Indus dolphin
Annual average discharge (m^3^/sec)	***	***	***
Flow accumulation (number of cells)	***	***	***
Hydrologically conditioned digital elevation model (m)	***	***	***
Stream gradient (decimeter/km)	***	***	***
Classical ordering of river (order)			
River flow corresponding to logarithmic size class (order)			
Strahler order (order)			
Distance to confluence (m)	***	***	***
River area (ha)	***	***	***
River volume (million m^3^)	***	***	***
Depth (m)			
Sinuosity (m)			
Inundation extent—annual maximum (%)	***	***	***
Inundation extent—annual minimum (%)	***	***	***
Degree of regulation (%)		***	***
Irrigated area extent (%)		***	***

With regard to classical river order, it can be seen that the Amazon dolphin and the Ganges dolphin were found to prefer third‐order tributaries. However, the Indus dolphin was mostly seen to prefer the mainstem of the rivers as depicted by classical river order. With regard to river flow order by logarithmic size class, the Amazon dolphin distribution peaked at order 7, the Ganges dolphin distribution showed peaks at orders 4 and 7, while the Indus dolphin distribution peaked at order 3; this observation concurs with the species‐specific preference for average river discharge with the Indus dolphin showing preference for river stretches with higher discharge than the rest of the species. In terms of Strahler order, the Amazon dolphin distribution peaked at lower Strahler orders, the Ganges dolphin distribution at moderate Strahler orders, while the Indus dolphin distribution peaked at higher Strahler orders.

All three dolphin species showed higher occurrence near confluences with peaks around <1000 m from the confluence. With regard to river area, the Amazon dolphins were mostly distributed in relatively smaller sized river segments while Indus dolphins were mostly distributed in larger segments and the Ganges dolphins were mostly observed in medium‐sized river segments. The Indus dolphins were generally found in reaches with much higher river volume than the Ganges dolphins and the Amazon dolphins. The Amazon dolphins were distributed in a higher range of depths than the Ganges dolphins and the Indus dolphins. Besides, all dolphin species mostly preferred slightly sinuous river segments.

Dolphin occurrences exhibited large variations in terms of inundation extent. The Amazon dolphin did not show much distinguishable pattern in terms of seasonal inundation extent but showed higher occurrence in areas with lower extent of permanent inundation. This could be an indication of the Amazon dolphin's habitat utilization of “varzea” (areas adjacent to main rivers which are seasonally flooded) demonstrating the importance of floodplain habitats for the species (Martin & da Silva, 2004). The Ganges dolphins and the Indus dolphins have shown higher occurrence in areas with high extent of seasonal inundation that corresponds to the upstream migration (to lower order rivers) of these dolphins during the high flow periods. This phenomenon of upstream migration has been noted for both species (Bhaagat, 1999; Sinha & Kannan, 2014).

### 3.2 Impacts of hydrological alterations on dolphins

The Amazon dolphin was found to be distributed mostly in rivers with lower degrees of regulation while the Ganges dolphin and Indus dolphin showed occurrence in rivers with moderate to higher degrees of regulation, respectively (Figure 2, Table 3). This is an indication of the fact that larger proportions of the Ganges dolphin and the Indus dolphin habitats have undergone hydrologic alteration and pockets of habitats with the occurrence of these dolphins represent shrinking subset of the former range of these two dolphin species compared with the Amazon dolphin. Likewise, the occurrences were mostly observed in rivers with lower irrigated area extent for the Amazon dolphin, in rivers with moderate irrigated area extent for the Indus dolphin, while the Ganges dolphin occurrence was observed in a wide range of irrigated area extents.

A total of 147 specific cases of hydrological alterations were assessed in this study with the largest number of alterations observed in the form of barrages followed by dams while alterations in the form of dredging, loop cutting, and spur dike were less. While all hydrologic alterations were caused by dams in the context of the Amazon dolphin, majority of the alterations in the distribution ranges of the Ganges dolphin and the Indus dolphin were barrages. The impacts of hydrological alterations were found to be predominantly negative with around 90% of the reported impacts (observed, speculated, or predicted) being negative. Of all the impacts included in the study, majority (65%) of the impacts were observed, 21% were speculated, and remaining 14% were predicted. Around 94% of all impacts were direct impacts. Upon the categorization of impacts caused by hydrological alterations into nine broad types, most impacts (35%) constituted habitat fragmentation followed by habitat reduction (24%); one instance of biological competition (with *Inia boliviensis*) was also noted. Habitat fragmentation refers to loss of connectivity of habitat patches, whereas habitat reduction refers to reduction in the overall extent of habitat of species. Highest number of impacts by hydrological alterations were noted for the Ganges dolphin followed by the Indus dolphin and the Amazon dolphin.

Impacts due to hydrological alterations have been evaluated in all the countries in the distribution range of the obligate freshwater dolphins. Majority of the impacts of hydrological alterations on the dolphins were reported from Pakistan (where majority of the Indus dolphin occurrences included in the study were reported), followed by India and Bangladesh. With respect to cross‐boundary impact, the highest number of impacts were noted in India, particularly along its border with Nepal that is traversed by the tributaries of the Ganges that drain the central Himalayas in Nepal. India is the only country encompassing the ranges of two extant species of obligate freshwater dolphins—the Ganges dolphin and the Indus dolphin; impacts of hydrological alterations have been noted for both species. In the Amazon dolphin range countries, the highest number of impacts was noted for Brazil. In terms of river basins, majority of the impacts were documented for the mainstem of the Indus river followed by the Upper Ganges and the Lower Ganges. In the South American rivers, most impacts were reported from the Madeira river. However, when considering tributaries, the Jamuna River, a tributary of the Ganges in Bangladesh, had the highest number of reported impacts.

For the Amazon dolphin, the impacts mostly relate to habitat fragmentation. Biological competition from other dolphin species due to hydrological alteration has also been predicted for this species, which is not the case for the Ganges dolphin and the Indus dolphin. This impact was predicted for an erroneous construction of fish bypass allowing the range expansion of *Inia geoffrensis* coming from Brazil affecting *Inia boliviensis* populations, which are already genetically vulnerable (Tavera et al., 2010). No positive impact of water development projects has been reported for the Amazon dolphin. In the case of the Ganges dolphin, the impacts have mostly been related to habitat reduction. Fishery overlap due to the water development has also been noted for this species but not for other dolphins. Hydrologic alterations have led to increase in the Ganges dolphin populations in several segments mostly due to increase in water depth. In the case of the Indus dolphin, the impacts are mostly related to habitat fragmentation. Positive impact has been associated with barrage on the Beas River enabling sustenance of a small Indus dolphin population (Momblanch et al., 2022).

Indirect impacts of hydrological alterations are also a matter of great concern as their cumulative impact further aggravates the precarious condition of the dolphin species facing multitude of threats. These impacts often manifest as ecological traps—the dolphins choose habitats based on their evolutionarily determined responses to environmental cues but they land instead on risky situations jeopardizing their survival. Likewise, increase in pollution was noted to have a negative impact on both the Amazon dolphin and the Ganges dolphin. For example, the Amazon dolphin habitats have seen increasing levels of mercury mostly due to mining activities (Trujillo, Crespo, et al., 2010), while in the case of the Ganges dolphin, the pollution is mostly linked to suspended solids and organotin compounds (Sinha, 2000).

## 4 DISCUSSION

### 4.1 Inference on habitat conditions observed in dolphin distribution areas

The occurrence of the Amazon dolphins mostly in third classical order of rivers and rivers with smaller average discharge (80% reported occurrence in 0–1000 m^3^/s) and smaller relative size (80% reported occurrence in 0–25 ha) indicates their distribution mostly in tributaries and smaller rivers. Among the Amazon dolphins, females are known to favor the flooded forest in order to meet the energy requirement for their young and for their safety (Martin & da Silva, 2004). Lateral hydrological connectivity that enables seasonal habitat use is considered a key determinant of their distribution. Hydrologic alterations such as those caused by dams that cutoff such a connectivity would pose a severe blow to the life cycle of the species.

The Ganges dolphins are mostly distributed in tributaries and moderate size rivers as shown by their occurrence in mostly third classical order of rivers, and rivers with small average discharge (80% reported occurrence in 0–500 m^3^/s) and moderate relative size (80% reported occurrence in 25–175 ha). The Ganges dolphins migrate to smaller tributaries during the monsoon season, which explains the second peak of distribution corresponding to rivers of seventh order in terms of logarithmic size order (Sinha & Kannan, 2014; Sinha & Sharma, 2003; Smith, 1993). Besides, the occurrence of the species in depression areas is in agreement with preference for deeper habitats near shallow depositional areas as reported by Kelkar (2008).

In contrast, the Indus dolphins are mostly distributed in mainstem mostly in one classical order of river and large size rivers (80% reported occurrence in 25–725 ha). The historical range of the Indus dolphin is fragmented into 17 river sections currently (Braulik et al., 2014). While their movement is not restricted to the mainstem of the Indus river and frequent movements to canals and other tributaries are observed, a large number of strandings occur when the canal gates are closed requiring rescue of the dolphins (Waqas et al., 2012).

As stated in our hypothesis we do see, similarity across the obligate freshwater dolphin species in the patterns of distribution in relation to hydrologic and physiographic parameters especially with variables such as river discharge, flow accumulation, distance to confluence, and sinuosity. However, we also observe variations across species in the influence of some other variables—for instance, in terms of Strahler river order, the Amazon dolphins prefer smaller order rivers, the Ganges dolphins prefer moderate order rivers while the Indus dolphins prefer higher order rivers.

### 4.2 Impact of hydrologic alterations from dams and other structures constructed or proposed in the distribution range of dolphins

Positive impacts of hydrologic alterations on dolphin distribution relate to the creation of suitable habitat conditions due to increase in countercurrent habitats, maintenance of minimum depth, increase in the number of deep pools, increase in river sinuosity, and increase in aggregate habitat (Bashir et al., 2010; Momblanch et al., 2022; Prajapati, 2021; Samad et al., 2022; Sinha, 2000; Smith, Aminul Haque, et al., 1998, Smith, Sinha, et al., 2000; Sonkar & Gaurav, 2020). Positive impacts on the Ganges dolphin and the Indus dolphin occurrence as a result of such alterations may be partly attributed to their ecological preference for greater depths. However, the Amazon dolphins are known to inhabit flooded forests. This preference especially reported for adult females and calves of the Amazon dolphins demonstrates the importance of lateral connectivity and floodplain habitats (Martin & da Silva, 2004). Morphological adaptation including flexible bodies, small dorsal fins, and large pectoral fins enables these dolphins to exploit narrow areas of habitat without getting stranded.

While some positive influence of hydrologic alterations on dolphin distributions have also been observed, the overall impact has been overwhelmingly negative. The nature of impact of such alterations on different species of dolphins might partly be attributable to the difference in ecological preference across species of obligate freshwater dolphins. Water‐related infrastructures and development projects threaten the unique biodiversity of large rivers. Water diversion, water abstraction, and changes in flow regime of rivers caused by hydraulic structures result in major constraints in habitat connectivity and resource availability for dolphins (Choudhary et al., 2012). Our study highlights the impact of such flow‐modifying structures in the form of habitat fragmentation and habitat reduction for the three extant species of obligate freshwater dolphin. In the context of more infrastructural development projects under construction and in planning across the distribution ranges of all obligate freshwater dolphin species including the interbasin water transfer mega‐projects National River Linking Project (NRLP, India) in the Ganges–Brahmaputra—Meghna basin and Brazil's large dams construction spree with at least 43 dams in Tapajo's Basin alone (Fearnside, 2015), hydrologic alterations will have an increasingly intensified impact on the long‐term survival and conservation of these species. Plans for development of water courses in the dolphin distribution ranges, for instance, plans for about 111 Inland National Waterways (NWs) spanning about 20,275 km for inland water transport including cargo and passenger/cruise vessels as per the National Waterways Act, 2016 in India add to the concerns of mounting pressures on dolphin distribution (Aggarwal et al., 2020).

Species such as the Amazon dolphin which are already under threat due to mercury pollution will be subject to heightened risk of biomagnification due to hydrologic alteration by dams. Damming for hydroelectricity generation in South America has been noted to favor mercury methylation in the reservoirs and is associated with biomagnification of mercury along trophic chains including a variety of fish in the reservoirs and downstream reaches of dams (Pestana et al., 2019). Hence, hydrological alterations will also aggravate indirect impacts to the dolphins from stressors such as water pollution.

When hydrological alterations are planned, the following considerations should be taken into account:

- Closing off old distributary channels can provide benefits of creation of eddy countercurrents and increase in river depth (Smith, Aminul Haque, et al., 1998). Hence, the effect of these projects needs to be further explored for consideration as options for reviving dolphin habitats.
- The fishery impacts especially in water diversion zones and water abstraction reaches should be given serious attention as dolphins have to deal with dual impacts of reduced flows and declining prey availability in such conditions.
- Release of pollutants in the water, especially in water abstraction areas should be regulated as reduced flows decrease the dilution capacity of rivers (Sinha et al., 2000). In addition, withdrawal of surface water during the low‐flow and dry seasons should take into account the ecological requirements of large riverine species such as the dolphins.
- Embankments have already been shown to impact the Ganges dolphins through reduction in hydraulic complexity and lateral connectivity of river ecosystems, elimination of spawning habitat for fish, leading to extirpation of dolphins in some cases (Sinha et al., 2000; Smith et al., 2000). Embankments can be even more threatening for the Amazon dolphins, which are known to favor the flooded forest habitats (Martin & da Silva, 2004).
- Dam construction authorities in South America must factor in the impact of dams on the Amazon dolphins given their vulnerability to stressors particularly pollution (Trujillo, Portocarrero, et al., 2010). Environmental Impact Assessment (EIA) of water‐based infrastructure projects should account for the enhanced risks of biomagnification of mercury pollution brought about by hydrologic alterations.
- Both the Amazon dolphins and the Ganges dolphins showed preference for the second and third‐order rivers (classical river order). Hence, hydrologic alterations on such rivers should also be critically evaluated for potential detrimental impacts on these species.
- All three species of obligate freshwater dolphins were found in a wide range of stream gradient and river discharge values. Hence, basin‐scale development planning in the distribution ranges of the dolphins must take into consideration the existing and potential impact on the dolphins. A Cumulative Impact Assessment (CIA) approach will be instrumental to predict and manage basin/sub‐basin scale changes in flow regimes especially when multiple infrastructures that modify water courses such as dams, water diversion for irrigation, interbasin water transfer projects are proposed for a river system.

### 4.3 Methodological considerations and limitations

In spite of a comprehensive data compilation effort, the study is associated with some limitations. Some river segments such as the Sundarbans Delta are not finely represented. Hence, many parts of these segments lack values of environmental variables. Likewise, the water depth presented in the study are approximations of river depths based on global river discharge estimates and simple hydraulic geometry laws (Linke et al., 2019). These do not exhibit actual hydraulic extremes found in the rivers. For instance. at its deepest point, the Amazon river is estimated to be 100 m deep. But the maximum depth observed in this study is just over 25 m, which points to a lack of fine‐scale hydrologic data.

Another limitation concerns the determination of the distance from nearest confluence for the cells in the rivers. The distance to confluence from a point that falls in the same raster cell as the confluence is calculated as 0. The value of 0 for distance to confluence should not be taken at face value as the locations of dolphins can be anywhere in the 30 arc second resolution (which corresponds to resolution of 1000 m × 1000 m) within the raster cell containing the confluence. To deal with this issue, we have classified distance to confluence less than 1000 m as “<1000” while all other values are continuous.

Although efforts were made to collect as much occurrence records as possible, there are areas where the dolphin occurrence records are lacking. For instance, records of the Ganges dolphin in the Ghaghara river, India, were not available to us. Besides, most of the occurrence records of the Amazon dolphins were obtained from tagged dolphins (Mosquera‐Guerra et al., 2021). These represent concentrated records of the species in specific rivers, which tends to over‐represent the range of variables in those segments.

## 5 CONCLUSION

The distribution of obligate freshwater dolphins is dependent on physiographic and hydrologic complexities of the rivers. Overall, it is observed that the Amazon dolphin is mostly distributed in tributaries and rivers with smaller average discharge, and smaller relative size. Lateral hydrological connectivity enables seasonal habitat use in the flooded forest habitats and this is considered a key determinant of its distribution. The Ganges dolphin is mostly distributed in tributaries and rivers with smaller average discharge and moderate relative size. Besides, the species had occurrence records in depression areas in the rivers. In contrast, the Indus dolphin is mostly distributed in mainstem and large size rivers. The negative impacts of hydrologic alteration, especially damming heavily outweigh the positive impacts on dolphin distribution. All three extant species of obligate freshwater dolphins are vulnerable to alterations in hydrologic regimes and habitat degradation and fragmentation caused by development of flow‐modifying infrastructure. As more new large‐scale hydrologic modifications are in construction and planning in the distribution ranges of these dolphins, such changes will further intensify the pressures on these endangered species. and will pose more severe challenge for their conservation. A basin‐scale outlook and consideration of the salient ecological requirements of these dolphins are imperative in the planning of water‐related infrastructures such as hydropower dams and water diversion to provide these species of freshwater megafauna a chance for long‐term survival in the face of mounting pressures from a multitude of stressors.

## AUTHOR CONTRIBUTIONS

Anu Rai was involved in conceptualization, methodology, software, formal analysis, investigation, data curation, writing—original draft preparation, writing—review and editing, visualization, and project administration. Tawqir Bashir was conceptualization, methodology, investigation, data curation, resources, writing—original draft preparation, writing—review and editing, and supervision. Elio Guarionex Lagunes – Díaz was involved in methodology, software, validation, visualization, and writing—review and editing. Bibek Shrestha was involved in investigation, and writing—review and editing.

## ACKNOWLEDGMENTS

We would like to thank Linke et al. (2019) for creating a data compendium on hydro‐environmental variables and making it open access without which the research could not be conducted.

## CONFLICT OF INTEREST STATEMENT

No competing interests.

## Supporting information


Appendix S1.

## Data Availability

The references of the occurrences record have been provided in Annex. The data used for analysis is available from HydroSHEDS website (https://www.hydrosheds.org/).
