# Supplementary material for: The effect of physiographic and hydrologic complexities and their alterations on the distribution of obligate freshwater dolphins
Source: Ecol Evol. 2023 May 21;13(5):e10106. doi: 10.1002/ece3.10106 (PMC10200689; doi:10.1002/ece3.10106)
Supplement: Supplementary file 1 — Appendix S1. [file ECE3-13-e10106-s001.docx]

**Annex I: Bibliography of occurrence records used in the study**

Alam, S.M.I., Hossain, M.M., Baki, M.A., Bhouiyan, N.A. (2015) Status of Ganges dolphin, Platanista gangetica gangetica (Roxburgh, 1801) in the river Buriganga, Dhaka. *Bangladesh Journal of Zoology*. **43**(1), 109–120.

Aziz, M.A. (2019) Atlas on Ganges River Dolphin and Irrawaddy Dolphin of Bangladesh. , 47.

Baki, M.A., Bhouiyan, N.A., Islam, Md.S., Alam, S.M.I., Shil, S., Hossain, Md.M. (2017) Present Status of Ganges River Dolphins *Platanista gangetica gangetica* (Roxburgh, 1801) in the Turag River, Dhaka, Bangladesh. *International Journal of Zoology*. **2017**, 1–7.

Campbell, E. (2015). *River Dolphin Population Assessment in Yarina Lagoon, Peru*.

Campbell, E. C., Alfaro Shigueto, J., Godley, B., & Mangel, J. (2017). Abundance estimate of the Amazon River dolphin (Inia geoffrensis) and the tucuxi (Sotalia fluviatilis) in southern Ucayali, Peru. *Latin American Journal of Aquatic Research*, *45*(5), 957–969. https://doi.org/10.3856/vol45-issue5-fulltext-11

Chase, I.C. (2019) *Annexure 6.3 : Report on Ganges River - Dolphin Population Census Survey in Brahmaputra River System*. The World Bank.

Chatterjee, A., Bhutia, P.T., Sen, A., Das, D., Mitra, P., De, J.K. (2015) The Status of the Ganges River Dolphin, Platanista Gangetica Gangetica (Roxburgh, 1801) in Coochbehar District of West Bengal, India.

Chowdhury, M.R., Mitra, S., Sen, S. (2016) On the Behaviour, abundance, habitat use and potential threats of the Gangetic Dolphin Platanista gangetica in southern West Bengal, India. *Journal of Threatened Taxa*. **8**(9), 9131–9137.

ENVIS Center. (2014). *Indus River Dolphin (Planista Gangetica minor) Status & Conservation in Punjab (India).pdf*.

Gravena, W., da Silva, V. M. F., da Silva, M. N. F., Farias, I. P., & Hrbek, T. (2015). Living between rapids: Genetic structure and hybridization in botos (Cetacea: Iniidae: *Inia* spp.) of the Madeira River, Brazil: Structure and hybridization in *Inia* spp. *Biological Journal of the Linnean Society*, *114*(4), 764–777. https://doi.org/10.1111/bij.12463

IUCN (2017) Platanista gangetica: Braulik, G.T. & Smith, B.D.: The IUCN Red List of Threatened Species 2019: e.T41758A151913336.

Khatri, T.B., Shah, D.N., Mishra, N. (2010) Post-flood status of the Endangered Ganges River Dolphin Platanista gangetica gangetica (Cetartiodactyla: Platanistidae) in the Koshi River, Nepal. *Journal of Threatened Taxa*, 1365–1371.

Mitra, S., Chowdhury, M.R. (2018) Possible range decline of Ganges River Dolphin Platanista gangetica (Mammalia: Cetartiodactyla: Platanistidae) in Indian Sundarban. *Journal of Threatened Taxa*. **10**(13), 12738–12748.

Mosquera-Guerra, F., Trujillo, F., Oliveira-da-Costa, M., Marmontel, M., Van Damme, P., Franco, N., Córdova, L., Campbell, E., Alfaro-Shigueto, J., Mena, J., Mangel, J., Oviedo, J., Carvajal-Castro, J., Mantilla-Meluk, H., & Armenteras-Pascual, D. (2021). Home range and movements of Amazon river dolphins Inia geoffrensis in the Amazon and Orinoco river basins. *Endangered Species Research*, *45*, 269–282. https://doi.org/10.3354/esr01133

Paudel, S. (2015) Status and Distribution of Ganges River Dolphin (Platanista Gangetica Gangetica) and Gharial Crocodile (Gavialis Gangeticus) in Nepal.

Sharma, G. (2013) Zoological Survey ofIndia Rio Gangetic Plains Regional Centre Sector - 8, Vijay Nagar, Patna - 800026. , 21.

Singh, H., Behera, S.K. (2018) Status of the Important Bioresources of Girwa River with Special Reference to Ganges River Dolphin (Platanista gangetica gangetica) in Katerniaghat Wildlife Sanctuary, Uttar Pradesh, India. In *Biological Resources of Water*.

Sinha, R.K. (1997) Status and conservation of Ganges River dolphin in Bhagirathi-Hooghly River systems in India. *International Journal of Ecology and Environmental Sciences*. **23**(4), 343–355.

Sinha, R.K., Sharma, G. (2003) Current status of the Ganges River dolphin, Platanista gangetica in the rivers Kosi and Son, Bihar, India. *J Bombay Nat Hist Soc*. **100**(1), 27–37.

Smith, B.D., Braulik, G., Strindberg, S., Ahmed, B., Mansur, R. (2006) Abundance of Irrawaddy Dolphins (Orcaella Brevirostris) and Ganges River Dolphins (Platanista Gangetica Gangetica) Estimated Using Concurrent Counts Made by Independent Teams in Waterways of the Sundarbans Mangrove Forest in Bangladesh. *Marine Mammal Science*. **22**(3), 527–547.

Wakid, A. (2005) *Conservation of Gangetic Dolphin in Brahmaputra River System, India*.

Wakid, A. (2009) Status and distribution of the endangered Gangetic dolphin (Platanista gangetica gangetica) in the Brahmaputra River within India in 2005. *CURRENT SCIENCE*. **97**(8), 9.

**Annex II: Bibliography of articles collected for data compilation**

Aisha, H., Braulik, G. T., Khan, U., Leslie, A., & Nawaz, R. (2017). Indus River Dolphin (Platanista gangetica minor)-an update on the current population assessment and conservation challenges. *IWC (International Whaling Commission)*, *10*.

Andrade, A. K. (2021). Estimativa populacional e distribuição do boto-vermelho (Inia geoffrensis) no reservatório da Usina Hidrelétrica de Balbina, Amazonas, Brasil.

Aziz, M. A. (2019). Conservation Action Plan for Ganges River Dolphin and Irrawaddy Dolphin of Bangladesh.

Baruah, D., Hazarika, L. P., Bakalial, B., Borah, S., Dutta, R., & Biswas, S. P. (2012). A grave danger for the Ganges dolphin (Platanista gangetica Roxburgh) in the Subansiri River due to a large hydroelectric project. *The Environmentalist*, *32*(1), 85–90. https://doi.org/10.1007/s10669-011-9375-0

Bashir, T., Khan, A., Behera, S. K., & Gautam, P. (2012). Factors determining occupancy of Ganges River dolphin (Platanista gangetica gangetica) during differing river discharges in the upper Ganges, India. *Mammalia*, *76*(4), 417–426. <https://doi.org/10.1515/mammalia-2011-0129>

Bashir, T., Khan, A., Gautam, P., & Behera, S. K. (2010). Abundance and Prey Availability Assessment of Ganges River Dolphin (Platanista gangetica gangetica) in a Stretch of Upper Ganges River, India. *Aquatic Mammals*, *36*(1), 19–26. https://doi.org/10.1578/AM.36.1.2010.19

Behera, S. K., Singh, H., & Sagar, V. (2013). Status of Ganges River Dolphin (*Platanista gangetica gangetica* ) in the Ganga River Basin, India: A review. *Aquatic Ecosystem Health & Management*, *16*(4), 425–432. https://doi.org/10.1080/14634988.2013.845069

Bhaagat, H. B. (2002). *Rescues of Indus Blind Dolphin (Platanista minor) in River Indus (Pakistan). Tigerpaper, 29*(3).

Braulik, G. T. (2006). Status assessment of the Indus River dolphin, Platanista gangetica minor, March–April 2001. *Biological Conservation*, *129*(4), 579–590. https://doi.org/10.1016/j.biocon.2005.11.026

Braulik, G. T. (2012). *Conservation ecology and phylogenetics of the Indus River dolphin (Platanista gangetica minor)* [Thesis, University of St Andrews]. https://research-repository.st-andrews.ac.uk/handle/10023/3036

Braulik, G. T., Arshad, M., Noureen, U., & Northridge, S. P. (2014). Habitat Fragmentation and Species Extirpation in Freshwater Ecosystems; Causes of Range Decline of the Indus River Dolphin (Platanista gangetica minor). *PLOS ONE*, *9*(7), e101657. <https://doi.org/10.1371/journal.pone.0101657>

Choudhary, S., Dey, S., Dey, S., Sagar, V., Nair, T., & Kelkar, N. (2012). River dolphin distribution in regulated river systems: Implications for dry-season flow regimes in the Gangetic basin: River Dolphin Distribution In Regulated River Systems. *Aquatic Conservation: Marine and Freshwater Ecosystems*, *22*(1), 11–25. <https://doi.org/10.1002/aqc.1240>

Gachal, G. S., & Slater, F. M. (2002). A Holistic Approach to the Conservation of the Indus River Dolphin (Platanista Minor) (Owen, 1853). *Biological Sciences - PJSIR*, *45*(1), 1.

Gaur, A., Akolkar, P., & Arora, M. P. (2009). *Water quality assessment of River Ganga for conservation of Gangetic dolphins (Platanista gangetica) at Garhmukteshwar*. 6.

Gravena, W., Farias, I. P., da Silva, M. N., da Silva, V. M., & Hrbek, T. (2014). Looking to the past and the future: were the Madeira River rapids a geographical barrier to the boto (Cetacea: Iniidae)?. *Conservation Genetics*, *15*(3), 619-629.

Karim, Md. M., & Bindra, N. (2016). Cumulative impact assessment for Sindh barrages. *Impact Assessment and Project Appraisal*, *34*(4), 346–358. https://doi.org/10.1080/14615517.2016.1228341

Kelkar, N. (2016). *Digging Our Rivers’ Graves?* 33.

Khanal, G., Suryawanshi, K. R., Awasthi, K. D., Dhakal, M., Subedi, N., Nath, D., Kandel, R. C., & Kelkar, N. (2016). Irrigation demands aggravate fishing threats to river dolphins in Nepal. *Biological Conservation*, *204*, 386–393.

Kreb, D., Reeves, R. R., Thomas, P. O., Braulik, G. T., & Smith, B. D. (2010). Establishing protected areas for Asian freshwater cetaceans: Freshwater cetaceans as flagship species for integrated river conservation management. *Final Workshop Report. Yayasan Konservasi RASI, Samarinda*.

Marmontel, M., dos Santos Lima, D., Funi, C., dos Santos, V. F., & Oliveira-da-Costa, M. (2021). Unveiling the Conservation Status of Inia and Sotalia in the Brazilian Northeastern Amazon. *Aquatic Mammals*, *47*(4).

Momblanch, A., Kelkar, N., Braulik, G., Krishnaswamy, J., & Holman, I. P. (2022). Exploring trade-offs between SDGs for Indus River Dolphin conservation and human water security in the regulated Beas River, India. *Sustainability Science*, *17*(4), 1619–1637. <https://doi.org/10.1007/s11625-021-01026-6>

Pavanato, H. J., Melo-Santos, G., Lima, D. S., Portocarrero-Aya, M., Paschoalini, M., Mosquera, F., Trujillo, F., Meneses, R., Marmontel, M., & Maretti, C. (2016). Risks of dam construction for South American river dolphins: a case study of the Tapajós River. *Endangered species research*, *31*, 47-60.

Portocarrero-Aya, M., Ferrer, A., Lasso, C. A., Ruiz-García, M., Bolaños-Jiménez, J., & Caballero, S. (2010). Status, distribution and conservation of the river dolphins Inia geoffrensis and Sotalia spp. *Venezuela. In: Trujillo F, Crespo E, van Damme PA, Usma JS (Eds) The Action Plan for South American River Dolphins*, *2020*, 17–28.

Prajapati, S. (2021). Stranding cases of endangered Ganges river dolphins in the Ghaghara–Sharada irrigation canals, Ganges river basin, India: Conservation implications. *Mammalia*, *85*(1), 39–46. <https://doi.org/10.1515/mammalia>

Reeves, R. R., & Chaudhry, A. A. (1998). Status of the Indus River dolphin Platanista minor. *Oryx*, *32*(1), 35–44. https://doi.org/10.1046/j.1365-3008.1998.00016.x

Reeves, R. R., & Leatherwood, S. (1994). Dams and River Dolphins: Can They Co-Exist? *Ambio*, *23*(3), 172–175.

Reeves, R. R., Chaudhry, A. A., & Khalid, U. (1991). Competing for Water on the Indus Plain: Is There a Future for Pakistan’s River Dolphins? *Environmental Conservation*, *18*(4), 341–350. <https://doi.org/10.1017/S0376892900022591>

Samad, I., Kelkar, N., & Krishnaswamy, J. (2022). Life at the borderline: Responses of Ganges river dolphins to dry-season flow regulation of river and canal habitats by the Farakka barrage. *Aquatic Conservation: Marine and Freshwater Ecosystems*, *32*(2), 294–308. https://doi.org/10.1002/aqc.3763

Silva, V. M. F. D., & Martin, A. R. (2010). Status, threats, conservation initiatives and possible solutions for Inia geoffrensis and Sotalia fluviatilis in Brazil. *The Action Plan for South American River Dolphins, pgs. 123-144*.

Sinha, R. K. (2000). Status of the Ganges River dolphins Platanista gangetica in the vicinity of Farakka Barrage, India. *Biology and conservation of freshwater cetaceans in Asia*, 42-48.

Sinha, R. K., & Kannan, K. (2014). Ganges River Dolphin: An Overview of Biology, Ecology, and Conservation Status in India. *AMBIO*, *43*(8), 1029–1046. <https://doi.org/10.1007/s13280-014-0534-7>

Sinha, R. K., & Sharma, G. (2003). Current status of the Ganges river dolphin, Platanista gangetica in the rivers Kosi and Son, Bihar, India. *J Bombay Nat Hist Soc*, *100*(1), 27-37.

Sinha, R. K., Smith, B. D., Sharma, G., Prasad, G., Choudhury, B. C., Sapkota, K., Sharma, R.K. & Behera, S. K. (2000). Status and distribution of the Ganges susu(Platanista gangetica) in the Ganges River system of India and Nepal. *Occas. Pap. IUCN Species Survival Comm.*, *23*, 54-61.

Smith, A. M., & Smith, B. D. (1998). *Review of status and threats to river cetaceans and recommendations for their conservation*. *6*, 18.

Smith, B. D. (1993). 1990 Status and conservation of the Ganges River dolphin Platanista gangetica in the Karnali River, Nepal. *Biological Conservation*, *66*(3), 159–169. https://doi.org/10.1016/0006-3207(93)90002-I

Smith, B. D., Ahmed, B., Ali, M. E., & Braulik, G. (2001). Status of the Ganges river dolphin or shushuk Platanista gangetica in Kaptai Lake and the southern rivers of Bangladesh. *Oryx*, *35*(1), 61–72. https://doi.org/10.1046/j.1365-3008.2001.00153.x

Smith, B. D., Braulik, G., Strindberg, S., Mansur, R., Diyan, M. A. A., & Ahmed, B. (2009). Habitat selection of freshwater‐dependent cetaceans and the potential effects of declining freshwater flows and sea‐level rise in waterways of the Sundarbans mangrove forest, Bangladesh. *Aquatic Conservation: Marine and Freshwater Ecosystems*, *19*(2), 209-225.

Smith, B. D., Haque, A. K. M. A., Hossain, M. S., & Khan, A. (1998). PROFILE: River Dolphins in Bangladesh: Conservation and the Effects of Water Development. *Environmental Management*, *22*(3), 323–335. https://doi.org/10.1007/s002679900108

Smith, B. D., Sinha, R. K., & Regmi, U. (1994). Status of Ganges River dolphins (Platanista gangetica) in the Karnali, Mahakali, Narayani and Sapta Kosi rivers of Nepal and India in 1993. *Marine Mammal Science*, *10*(3), 368–375. <https://doi.org/10.1111/j.1748-7692.1994>

Smith, B. D., Sinha, R. K., Kaiya, Z., Chaudhry, A. A., Renjun, L., Ding, W., Ahmed, B., Haque, A.A., Mohan, R.S.L. & Sapkota, K. (2000). Register of water development projects affecting river cetaceans in Asia. *Biology and conservation of freshwater cetaceans in Asia*, *22*, 22-39.

Sonkar, G. K., & Gaurav, K. (2020). Assessing the impact of large barrages on habitat of the Ganga River dolphin. *River Research and Applications*, *36*(9), 1916–1931. https://doi.org/10.1002/rra.3715

Tavera, G., Aliaga-Rossel, E. R., Van Damme, P. A., & Crespo, A. (2010). Distribution and conservation status of the Bolivian river dolphin Inia boliviensis (d’Orbigny 1832). *The action plan for South American river dolphins*, *2020*, 99.

Trujillo, F., Portocarrero-Aya, M., Gomez-Salazar, C., Diazgranados, M. C., Castellanos-Mora, L., Ruiz-García, M., & Caballero, S. (2010). Status and conservation of river dolphins Inia geoffrensis and Sotalia fluviatilis in Colombia. *The Action Plan for South American River Dolphins*, *2020*, 99.

Utreras, V., Suarez, E., & Jalil, S. (2010). Inia geoffrensis and Sotalia fluviatilis: A brief review of the ecology and conservation status of river dolphins in the Ecuadorian Amazon. *The action plan for South American river dolphins*, *2020*, 59-81.

Waqas, U., Malik, M. I., & Khokhar, L. A. (2012). Conservation of Indus River Dolphin (Platanista gangetica minor) in the Indus River system, Pakistan: an overview. *Rec. Zool. Surv. Pak*, *21*, 82-85.
